# Chemical and Enantiomeric Profiling of *Melaleuca viridiflora* Sol. ex Gaertn. Essential Oil and Evaluation of Its Antimicrobial, Antioxidant, and Anticholinesterase Activities

**DOI:** 10.3390/plants15193029

**Published:** 2026-10-03

**Authors:** Eduardo Valarezo, Jennifer Herrera-Rodríguez, Daniela Berrú-Celi, James Calva, Ximena Jaramillo-Fierro, Miguel Angel Meneses

**Affiliations:** 1 Departamento de Química y Producción, Universidad Técnica Particular de Loja, Loja 1101608, Ecuador; jwcalva@utpl.edu.ec (J.C.); xvjaramillo@utpl.edu.ec (X.J.-F.); mameneses@utpl.edu.ec (M.A.M.); 2 Carrera de Bioquímica y Farmacia, Universidad Técnica Particular de Loja, Loja 1101608, Ecuador; jmherrera14@utpl.edu.ec (J.H.-R.); djberru@utpl.edu.ec (D.B.-C.)

**Keywords:** Ecuador, Myrtaceae, physical properties, GC-MS, GC-FID

## Abstract

*Melaleuca viridiflora* (niaouli) is an Australian species that has been introduced into different regions worldwide because of its broad adaptability and potential applications. This work aimed to characterize the chemical and enantiomeric composition of the leaf essential oil from *M. viridiflora* cultivated in the Americas and to explore its biological properties. The essential oil was isolated by steam distillation and chemically characterized using gas chromatographic techniques, while its enantiomeric profile was established using two chiral stationary phases. Biological activity was investigated by determining the antimicrobial effects against a panel of Gram-positive and Gram-negative bacteria, *Candida albicans*, and *Aspergillus niger* using the broth microdilution assay. In addition, the antioxidant capacity was examined by ABTS and DPPH radical-scavenging assays, and acetylcholinesterase inhibitory activity was determined by a spectrophotometric method. Fifty compounds were identified, accounting for 99.07% of the total composition. Sesquiterpene hydrocarbons represented the largest group in terms of number of compounds (16), whereas oxygenated monoterpenes predominated in relative abundance (45.27%). The major constituents were 1,8-cineole (35.66 ± 0.37%), viridiflorol (14.20 ± 0.12%), limonene (11.04 ± 0.06%), *α*-terpineol (7.89 ± 0.71%), and *α*-pinene (7.42 ± 0.11%). Six pairs of enantiomers were identified. Overall, the essential oil displayed a biological profile characterized by limited antimicrobial and antioxidant effects but a comparatively more pronounced anticholinesterase activity. Antimicrobial effects were observed at 4000 μg/mL against *Listeria monocytogenes*, *Enterococcus faecium*, and *Aspergillus niger*, while antioxidant activity was weak, with an ABTS SC_50_ of 1.2 mg/mL. In contrast, the oil exhibited moderate anticholinesterase activity, with an IC_50_ of 65.98 ± 1.0 μg/mL. Taken together, these findings indicate that the biological profile of *M. viridiflora* essential oil is primarily distinguished by its anticholinesterase activity rather than by antimicrobial or antioxidant effects. This study provides the first report for *Melaleuca viridiflora* cultivated in the Americas.

## 1. Introduction

Essential oils (EOs) are volatile and complex mixtures of low-molecular-weight secondary metabolites, predominantly terpenes and their oxygenated derivatives, biosynthesized in specialized plant tissues [1]. They are produced in various organs, including leaves, flowers, bark, fruits, seeds, stems, and roots, and stored in secretory structures such as glands, ducts, cavities, or glandular trichomes [2]. These metabolites are responsible for the characteristic aroma of numerous plant species distributed across diverse botanical families, particularly Apiaceae, Asteraceae, Burseraceae, Cupressaceae, Lamiaceae, Lauraceae, Leguminosae, Myrtaceae, Pinaceae, Piperaceae, Rutaceae and Zingiberaceae [3]. Beyond their ecological roles in plant defense and communication, essential oils have long been used in traditional medicine, food preservation, and perfumery. Their documented antimicrobial, antifungal, antiviral, antiparasitic, antioxidant, and insecticidal properties have promoted their incorporation into pharmaceutical, cosmetic, agricultural, and food systems [4]. Consequently, increasing scientific attention has been directed toward elucidating their chemical profiles, stereochemical composition, and biological activities, particularly in the context of natural product-based alternatives to synthetic compounds.

Myrtaceae Juss. is a family of evergreen, aromatic, shrubby, and arboreal dicotyledonous plants belonging to the order Myrtales. The myrtaceae family includes approximately 127 genera and 6547 species [5]. Myrtaceae are widely distributed throughout the tropical and subtropical regions of the Americas, Africa, Asia, and Australia. They thrive in diverse terrestrial ecosystems, except for arid and semi-arid zones [6]. Most myrtaceae have high economic value due to the presence of species with edible fruits and raw materials for obtaining EOs, spices, and timber. Likewise, several species are cultivated for their ornamental value [7]. Members of this family are characterized by the presence of secretory cavities rich in essential oils, as well as by their aromatic leaves and economic importance. EOs of several genera such as *Eucalyptus, Eugenia, Leptospermum, Melaleuca, Myrtus* and *Pimenta* have been studied due to their phytochemical composition and bioactive properties [3].

Within Myrtaceae, the genus *Melaleuca* L. includes 391 species native to Australia [8], although many species have been introduced and cultivated in other regions, such as the southern parts of North America, southeastern America, southeast Asia, southern Africa and Europe, for ornamental, medicinal, and industrial purposes [9]. The name *Melaleuca* originates from the Greek words *melas*, meaning black or dark, and *leucon*, meaning white. This designation refers to the contrast observed in the first described species, *M. leucadendra*, which is characterized by its white branches and a dark trunk that is often blackened because of fire exposure [10]. Plants of this genus are commonly known as paperbark trees or tea trees and are characterized by evergreen shrubs or small trees with aromatic leaves rich in essential oils. The essential oils obtained from *Melaleuca* species are used in traditional medicine and modern pharmacology because of their antimicrobial, anti-inflammatory, antioxidant, and insecticidal activities [11]. 

**Figure 1:**
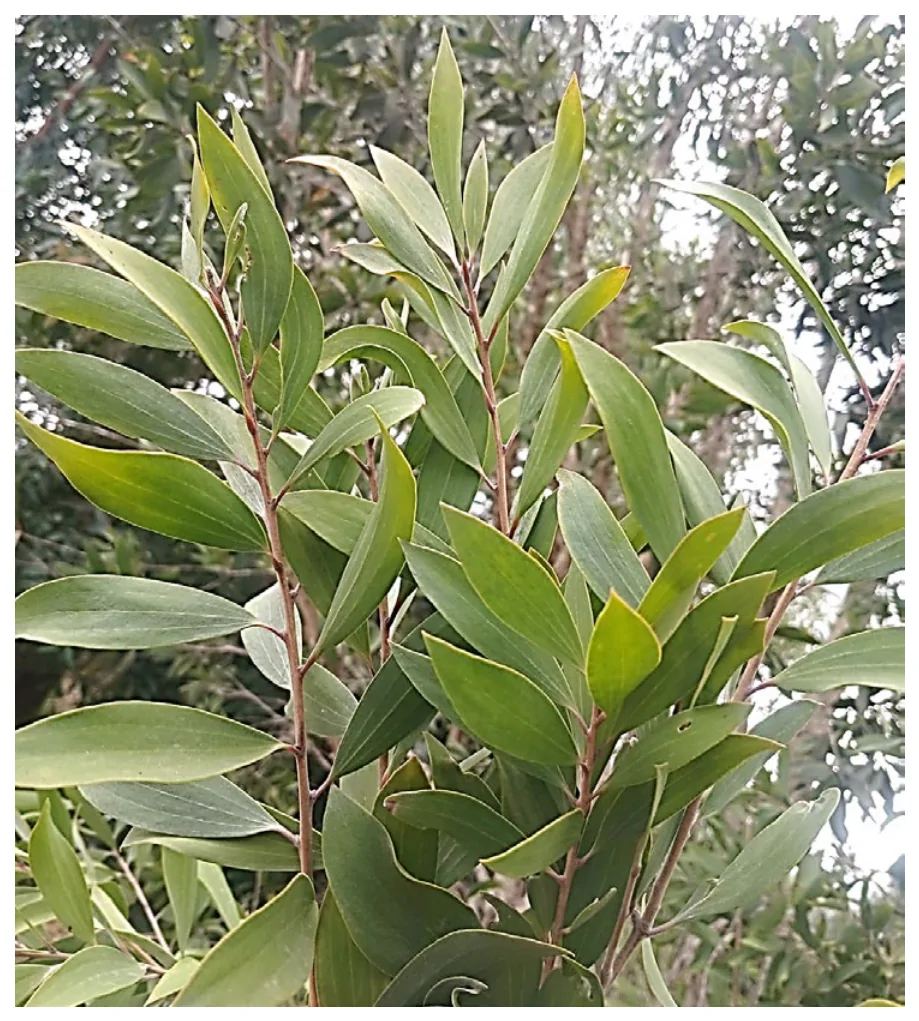
Species *Melaleuca viridiflora* Sol. ex Gaertn.

*Melaleuca viridiflora* Sol. ex Gaertn., commonly known as paperbark or niaouli (New Caledonia) [12], is described as a shrub or tree ranging from 5 to 10 m in height, occasionally reaching up to 25 m, with papery bark that varies in color from white to light brown or grey. The branchlets are generally glabrescent and silky. Leaves are alternate, long-petiolate, and narrowly elliptic to ovate, measuring 70–195 mm in length and 19–76 mm in width, with an attenuate base and acute to obtuse or emarginate apex (Figure 1). They show longitudinal venation with 5–9 veins and possess dense or moderately dense, though often obscure, oil glands. The inflorescences are spicate and pseudoterminal, sometimes also occurring in the upper axils, with 8–25 triads and a width of up to 55 mm. Floral structures include deciduous petals and stamens grouped in bundles, with filaments that vary in color from greenish to cream, white, or red. The fruit is small, measuring 2.3–5.5 mm in length, with deciduous calyx lobes and obvolute cotyledons. This species is naturally distributed across northern Australia, including Western Australia, the Northern Territory, and Queensland, as well as parts of Indonesia and Papua New Guinea. It grows in a wide range of habitats, such as closed woodland, heathlands, seasonal swamps, and areas near water bodies, adapting to diverse soil types including sandy, clay, lateritic, basaltic, and rocky substrates [10]. Due to its adaptability, *M. viridiflora* has also been cultivated in other regions of the world. In Ecuador, this species has the status of introduced.

The genus Melaleuca (Myrtaceae) includes many essential oil-producing species of recognized economic importance. Nevertheless, research has largely centered on *Melaleuca alternifolia* [13], while other species remain comparatively underexplored. In the case of *M. viridiflora*, available information about its essential oil—especially concerning physicochemical parameters, enantiomeric distribution, and biological activities—is still limited, particularly when the species is cultivated outside its native habitat. Therefore, this study aims to isolate and characterize the essential oil obtained from the leaves of *M. viridiflora*, including the determination of its physicochemical properties, chemical composition, and enantiomeric distribution. Furthermore, its antimicrobial, antifungal, antioxidant, and anticholinesterase activities were evaluated to explore its potential bioactive profile. Leaves were selected for essential oil characterization because they constitute a renewable and available plant material and are commonly used for EO extraction and chemical characterization in vascular plant species. Their use also facilitates comparison with previously reported chemical profiles. Therefore, characterization of the leaf essential oil of *M. viridiflora* provides baseline information for subsequent comparative studies addressing chemical and biological variability among different plant organs. It is hypothesized that the essential oil of this species contains terpenoids characteristic of the genus *Melaleuca* [14] and shows significant biological activities that may support its prospective applications in pharmaceutical, cosmetic, and food-related industries.

## 2. Results

### 2.1. Essential Oil Yield

Approximately 7 kg of fresh leaves of *M. viridiflora* were steam distilled in three different batches for the purpose of isolating the EO. From this plant material, 17.1 mL of EO was obtained, corresponding to a yield of 0.245 ± 0.03% (*v*/*w*) or 2.45 ± 0.3 mL/kg.

### 2.2. Physical Properties of Essential Oil

The essential oil obtained from the leaves of *M. viridiflora* was a moderately yellow, oily liquid with a density lower than that of water. The mean values of its physical properties are summarized in Table 1.

### 2.3. Chemical Composition of Essential Oil

**Figure 2:**
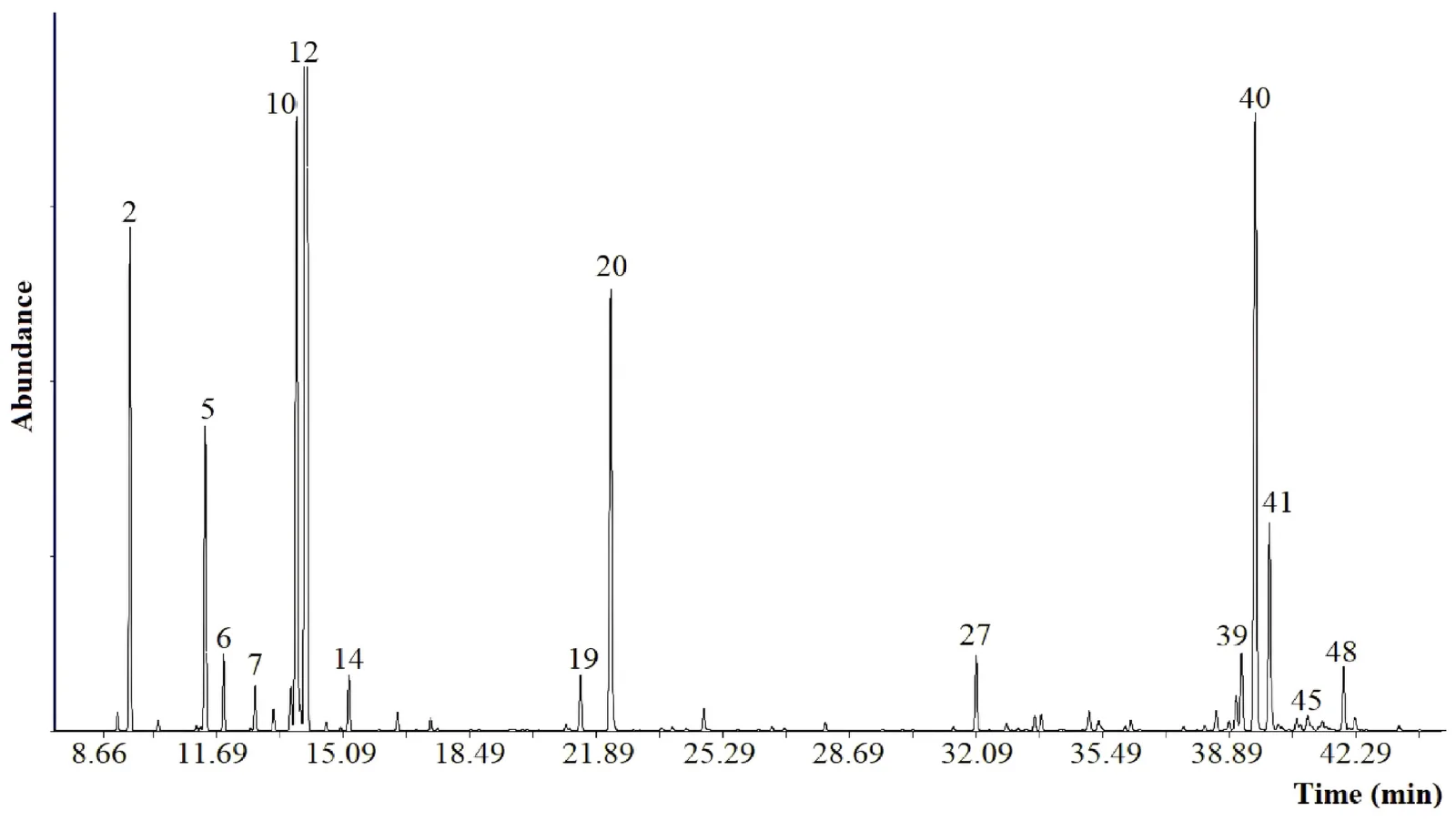
Representative chromatograms of the essential oil of *Melaleuca viridiflora*; the numbers correspond to the compound numbers (CN) in Table 2.

Using gas chromatography coupled with mass spectrometry (GC-MS) and gas chromatography equipped with a flame ionization detector (GC-FID), fifty compounds were identified in the essential oil obtained from *Melaleuca viridiflora*. Figure 2 shows a representative chromatograms of this EO. Table 2 summarizes the compound number (CN), retention time (RT) at which each compound eluted, calculated retention indices (RIC), retention indices reported in the literature (RIR), relative abundance (%) together with the standard deviation (SD), chemical classification (GC), molecular formula (CF), and monoisotopic mass (MM) of the identified constituents. Overall, the identified compounds represented 99.07% of the total composition of the essential oil, indicating a high level of characterization.

**Figure 3:**
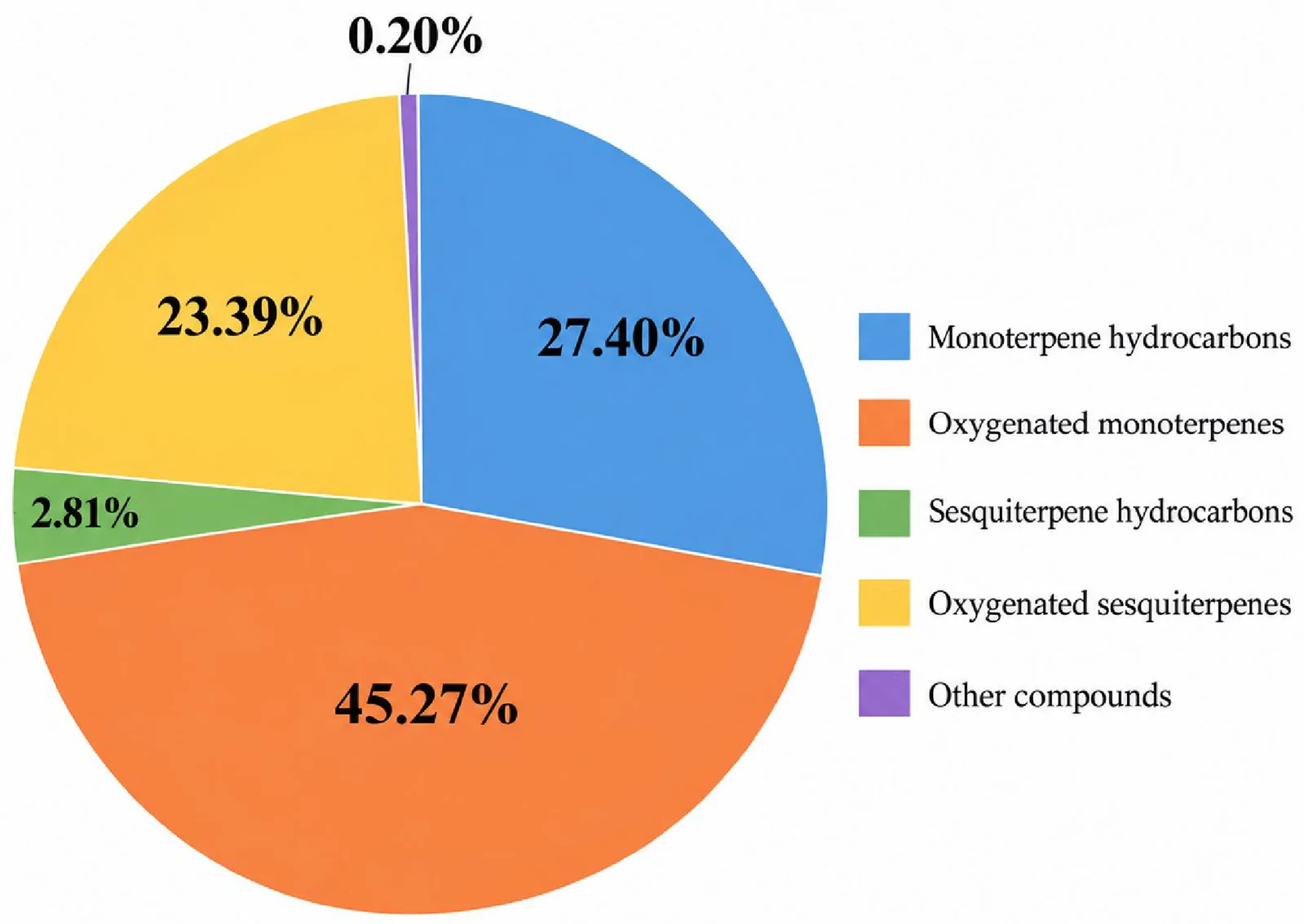
Grouping of compounds in the essential oil of *Melaleuca viridiflora*; total identified, 99.07%.

The identified constituents were grouped into five chemical classes according to their carbon skeleton (monoterpenes and sesquiterpenes), the presence or absence of oxygenated functional groups, and non-terpenic compounds. Oxygenated monoterpenes were the predominant group, accounting for 45.27% of the total oil (Figure 3). These results highlight the dominance of oxygenated compounds in the essential oil, which are often associated with enhanced biological activity and organoleptic properties.

Among the major constituents, 1,8-cineole (CN: 12), classified as an oxygenated monoterpene (CF: C_10_H_18_O; MM: 154.14 Da), was the most abundant compound, reaching 35.66 ± 0.37%. The second most abundant compound was viridiflorol (CN: 40), an oxygenated sesquiterpene (CF: C_15_H_26_O; MM: 222.20 Da), which accounted for 14.20 ± 0.12%. In addition, limonene (CN: 10), a monoterpene hydrocarbon (CF: C_10_H_16_; MM: 136.13 Da), represented 11.04 ± 0.06% of the oil, followed by *α*-terpineol (CN: 20), which reached 7.89 ± 0.71% and *α*-pinene (7.42 ± 0.11%). Other relevant constituents included *β*-pinene (4.58 ± 0.04%), belonging to the monoterpene hydrocarbons and ledol (4.08 ± 0.05%) contributed to the oxygenated sesquiterpene fraction.

### 2.4. Enantiomeric Distribution

Using MEGA-DEX DET Beta and MEGA-DEX DAC Beta enantioselective stationary phase columns, it was possible to separate six pairs of enantiomers in the EO from leaves of *M. viridiflora*. Rs values ranged from 1.1 to 1.6. Baseline resolution (Rs ≥ 1.5) was achieved for limonene and *α*-terpineol on the MEGA-DEX DET Beta column, whereas the remaining enantiomeric pairs showed partial resolution (Rs ≥ 1 and < 1.5). Table 3 shows the retention time (RT), retention index (RI), enantiomeric distribution (ED), enantiomeric excess (e.e.) and resolution (Rs) for each pair of compounds. Of the separated enantiomers, (−)-(1*S*,5*S*)-*β*-pinene was the most abundant with an enantiomeric excess of 94.67%, and *α*-terpineol was found in both columns.

### 2.5. Antibacterial Activity

The antibacterial activity of the EO of *M. viridiflora* was tested against four Gram-negative bacilli, one Gram-positive bacillus, and three Gram-positive cocci. Table 4 shows the minimum inhibitory concentration (MIC) values of the EO and the positive and negative control. The EO had weak activity against *Listeria monocytogenes* and *Enterococcus faecium* with a MIC of 4000 µg/mL. All microorganisms showed normal growth in negative control.

### 2.6. Antifungal Activity

The EO of *M. viridiflora* was tested against one fungus and a yeast, Table 5 shows the values for the MIC. The essential oil showed weak activity against *Aspergillus niger* with a MIC of 4000 µg/mL.

### 2.7. Antioxidant Activity

The free-radical scavenging potential of the EO of *M. viridiflora* was evaluated through the ABTS and DPPH assays. The resulting SC_50_ values, expressed as µg/mL, were determined for both the EO and the reference antioxidant. In addition, antioxidant activity was quantified as trolox equivalent antioxidant capacity (TEAC, µmol TE/g). These results are presented in Table 6.

### 2.8. Anticholinesterase Activity

**Figure 4:**
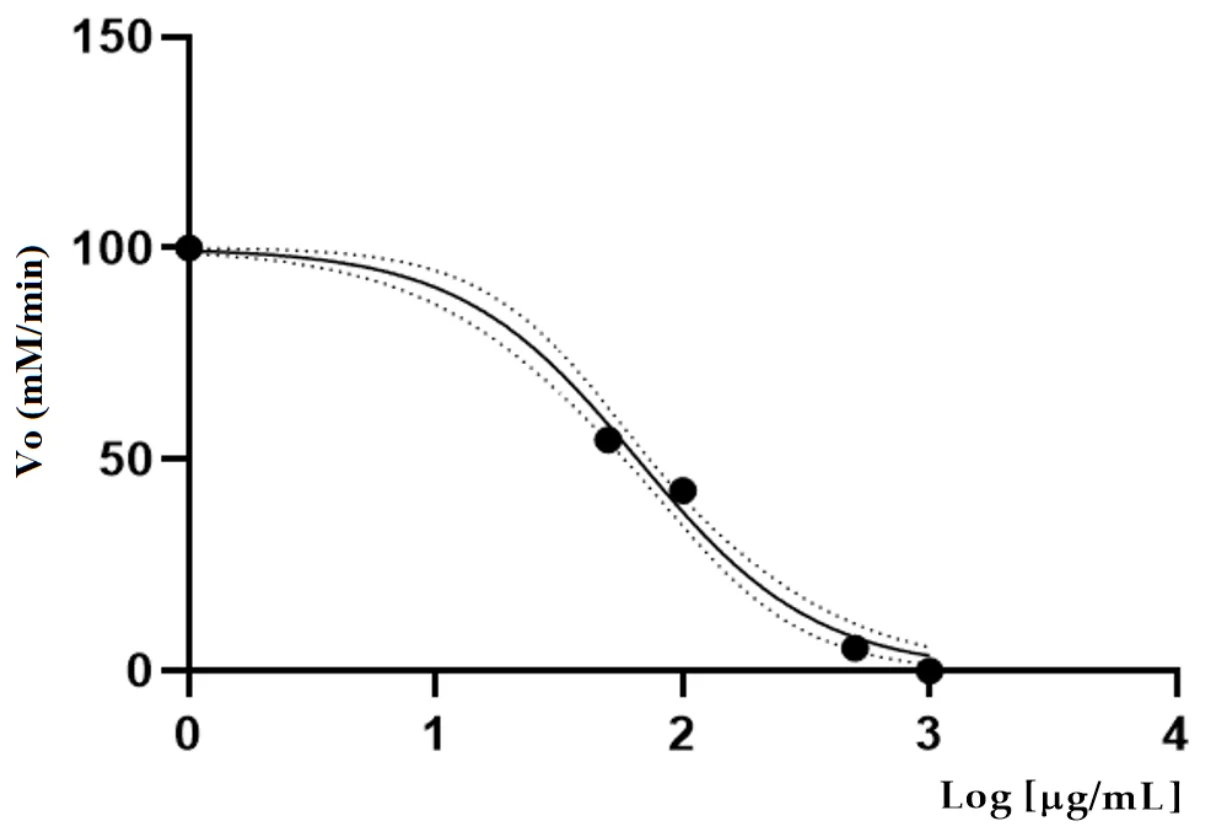
Anticholinesterase activity of essential oil from leaves of *Melaleuca viridiflora*.

The EO obtained from *M. viridiflora* leaves showed inhibitory activity against acetylcholinesterase (AChE), with an IC_50_ of 65.98 ± 1.0 µg/mL (Figure 4). Under the same assay conditions, donepezil, employed as the positive control, displayed an IC_50_ of 12.4 ± 1.4 µg/mL. Figure 1 shows the rate of the reaction curve for anticholinesterase (anti-AChE) activity.

## 3. Discussion

The essential oil yield from *M. viridiflora* leaves was 2.45 ± 0.3 mL/kg, which could be classified as low, according to the categorization for plant species proposed by the “Science and Technology for Development” (CYTED) (yield values in essential oil greater than 10 mL/Kg are considered high, values between 5 mL/Kg and 10 mL/Kg intermediate, and values less than 5 mL/Kg low) [15]. Species of the genus Melaleuca are known for their ability to produce essential oils with varying yields, depending on the species, the collection site, and the extraction method. For the Australian species *Melaleuca deanii*, essential oil yields obtained by hydrodistillation ranged from 0.27% to 1.79%, decreasing to 0.10% in a tree monitored for 46 years. For *Melaleuca hypericifolia*, yields ranged from 0.30% to 2.28% [16]. The species *Melaleuca quinquenervia*, collected in Cuba, yielded an average of 0.8% [17]. The yield for *Melaleuca cajuputi* ranged from 0.6% to 0.7% in fresh leaves collected in Vietnam [18]. Using conventional hydrodistillation, a yield of 0.2% to 1.02% was obtained, while with ultrasound-assisted extraction (UAE), a higher yield of 4.55% was achieved for the species *Melaleuca bracteata* [19,20]. In populations from New South Wales, Australia, for the species *Melaleuca ericifolia*, an average yield of 3.2% ± 0.95 was reported, while in other regions such as Tasmania, the yield was on average 0.6% ± 0.3% [21]. For the species *M. viridiflora*, Widiyanto and Siarudin reported yields of <0.1% (trees with red flowers) and 0.23% (trees with white flowers) in 2013 [22]. The Australian Government, through the Australian Centre for International Agricultural Research, reports yields ranging from 0.4% to 2.1% [23].

The physical properties of essential oils, including color, density, refractive index, and specific optical rotation, are due to their chemical composition and relative abundance of their volatile constituents. The essential oil obtained in this study exhibited a brass color (RGB: 185, 157, 50, Table 1). Besides serving as a descriptive characteristic, color represents an important quality attribute because it contributes to product standardization and influences consumer acceptance, particularly when essential oils are incorporated into food, cosmetic, or personal care formulations [24]. As expected for hydrophobic mixtures rich in terpenoids, the density of the essential oil was lower than water, a characteristic that facilitates phase separation during hydrodistillation. The essential oil extracted from *M. viridiflora* leaves presented a density of 0.8933 ± 0.0057 gcm^−3^. For this same species, specific density values between 0.830 and 0.838 were reported for oil extracted from species in Papua, Indonesia [22], and 0.8764 (Maryborough) and 0.8800 (Brisbane) for oil from Australia [25]. The refractive index is another relevant physicochemical parameter because it provides indirect information about the molecular composition of an essential oil. This property depends on the refractive indices of the individual constituents and their respective molar proportions within the mixture [26]. In the present study, the refractive index of the leaf essential oil was 1.4733 ± 0.0005, a value that agrees with the predominance of monoterpenes such as 1,8-cineole (1.448 to 1.490), limonene (1.470 to 1.485) and *α*-pinene (1.453 to 1.5053) [27]. For the essential oil *M. viridiflora* from Australia, indices of 1.4700 and 1.4719 were reported for species from Maryborough and Brisbane, respectively [25].

The essential oil of *M. viridiflora* exhibited an optical rotatory power of −6.48 ± 0.10°. Optical rotation is directly influenced by the presence of chiral compounds in the essential oil and the enantiomeric composition of these compounds. In this case, the low optical activity of the essential oil studied may correspond to the non-chiral nature of the main compound, 1,8-cineole. Although Fujita and Yasuji reported in 1956 that 1,8-cineole has an Optical Rotatory Power of +5.55° [28], some commercial companies (Thermo Fisher Scientific and Prodasynth) currently report that this pure compound has an optical rotation close to zero (between −0.3 and +0.3; measured at 20 °C, 589 nm). Considering that the EO contains main compounds like limonene, which are chiral, with (+)-limonene exhibiting a specific rotation of around +100° and (–)-limonene exhibiting a value of around −100° [27], it is clear that the specific optical rotation is influenced by the different enantiomers of chiral compounds, which rotate polarized light in different directions, making it difficult to attribute optical activity to a single compound. 

Melaleuca species are known for the diversity of aromatic constituents in their volatile oils. These include monoterpenoids, such as terpinen-4-ol, terpinolene, *p*-cymene, *α*-terpineol, 1,8-cineole, *α*-pinene, and *α*-terpinene; sesquiterpenoids, such as *β*-caryophyllene, ledol, (*E*)-nerolidol, and viridiflorol; and phenylpropanoids, such as methyl eugenol and methyl isoeugenol, which have been reported in tea tree oil [29,30]. Of this genus, the most studied species is *Melaleuca alternifolia*, commonly known as tea tree oil, with terpinen-4-ol being the main compound. Other significant compounds present in the oil include *α*-terpinene, *γ*-terpinene, *α*-terpineol, and 1,8-cineole (also known as eucalyptol), with their relative abundances varying depending on the region of plant harvest and environmental factors [31,32].

The phytochemical profiling of the EO obtained from leaves of *M. viridiflora* led to the identification of fifty constituents, representing 99.07% of the total oil composition. The chemical profile was dominated by oxygenated components, with oxygenated monoterpenes (45.27%), followed by oxygenated sesquiterpenes (23.39%), while the monoterpene hydrocarbons contributed 27.40%. The major constituent was 1,8-cineole (35.66 ± 0.37%). Based on the reviewed bibliography, it was not possible to find studies on the chemical composition of the essential oil of *M. viridiflora*. However, in a screening study of myrtaceous tree species for production of essential oils in northern Queensland carried out in 2007, it is reported that the main compound in *M. viridiflora* oil is methyl cinnamate with a range between 82% and 94% [33]. In an in vitro study of the antibacterial effect of exotic plant essential oils on the honeybee pathogen *Paenibacillus larvae* developed in Argentina in 2010 with commercial essential oils from Italy, it is reported that among the major compounds of the essential oil of this species are terpinen-4-ol (29.09%), *α*-pinene (21.63%), limonene (17.40%), *α*-terpineol (8.07%) and *β*-pinene (7.30%), and that 1,8-cineole only reaches 2.18% [34]. In a study on the behavioral effects of five plant essential oils on the green pea aphid acyrthosiphon pisum (Harris) (Homoptera: Aphididae) carried out in 2017 with essential oil extracted by hydrodistillation from leaves collected in Antananarivo, Madagascar between June and July 2008, it is reported that among the major compounds are 1,8-cineole with 55.54%, *α*-pinene (12.96%), elemol (9.89%) and terpinen-4-ol (8.43%) [35]. In a more recent study (2024) on the efficiency of *M. viridiflora* and other essential oils for *Varroa Mite* (Acari: Varroidae) control in honeybee (Hymenoptera: Apidae) colonies carried out in Turkey with commercial oil acquired from a local company, it is indicated that among the active ingredients are 1,8-cineole (31.5%), viridiflorol (21.7%), *α*-pinene (17.9%), *α*-terpineol (6.5%), terpinene 4-ol (2.6%), *γ*-terpinene (2.3%) and *β*-pinene (1.9%) [36]. The component of an essential oil can change because the chemical composition of essential oils can exhibit considerable variability, such that individuals of the same species can produce substantially different chemical profiles. This variability results from the interaction between genetic and environmental factors, including genotype, geographic origin, climatic and soil conditions, water availability, altitude, harvest time, phenological stage, and the plant part used, as well as factors related to the processing, extraction, and storage of the essential oil [37].

In previous studies, 1,8-cineole has been identified as the major or predominant component in the essential oils of several species of Melaleuca, among which are *Melaleuca atroviridis*, with a leaf oil in which 1,8-cineole constitutes 73.2% of the total oil, making it the main component by a significant margin [38]. *Melaleuca teretifolia* also has a chemotype with a very high 1,8-cineole content, reaching 84.0% in its EO [39]. *Melaleuca fulgens* is another species with a high proportion of 1,8-cineole (56.9%) [40]. *Melaleuca quinquenervia* shows considerable variability in 1,8-cineole content across different samples, with some samples having up to 76% 1,8-cineole, showing that it can be the predominant compound in certain chemotypes of this species [30]. *Melaleuca leucadendron* also contains 1,8-cineole as a major compound, although at a lower percentage (19.9%), so it may not always be the predominant compound but is still significant [41]. 

The enantiomeric profile of *M. viridiflora* EO showed predominantly non-racemic distributions, showing marked stereoselectivity in the biosynthesis of its chiral monoterpenes. The strongest enantiomeric differentiation was observed for *α*-pinene and *β*-pinene, with e.e. values of 96.61% and 94.67%, respectively. *α*-Pinene was almost exclusively represented by one enantiomer (98.31%), while (−)-(1*S*,5*S*)-*β*-pinene clearly predominated (97.33%) over (+)-(1*R*,5*R*)-*β*-pinene (2.67%). A comparable pattern has been reported within the Myrtaceae family. In *Myrcia mollis*, for example, (−)-*β*-pinene represented 97.1% of the enantiomeric distribution. In contrast, *Myrcianthes myrsinoides* showed a substantially less stereoselective β-pinene profile, with approximately 60% of (−)-*β*-pinene [42]. Lorenzo et al. [43] reported an almost racemic *β*-pinene distribution in Myrcianthes cisplatensis, with approximately 51% enantiomeric purity for (−)-*β*-pinene, despite much stronger enantiomeric preferences for other monoterpenes in the same oil, including (+)-*α*-pinene (96%) and (+)-limonene (100%). More broadly, enantioselective GC analyses of Myrtaceae essential oils have demonstrated that β-pinene and other chiral monoterpenes can exhibit markedly species-dependent enantiomeric distributions [44]. Limonene also exhibited an asymmetric distribution (77.92:22.08; e.e. = 55.85%), although less pronounced than that of the pinenes. Linalool analyzed on the MEGA-DEX DET Beta column was enantiomerically enriched, with (+)-(3*S*)-linalool accounting for 75.57% and (−)-(3*R*)-linalool for 24.43% (e.e. = 51.15%). Enantioselective profiling can provide an additional level of chemical information for essential oils because the relative proportions of stereoisomers may reflect species-specific biosynthetic processes and may therefore contribute to assessments of botanical origin and authenticity. Wong et al. [45] demonstrated this approach in 57 essential oil samples of *Melaleuca alternifolia* obtained from Australian plantations, which displayed highly reproducible enantiomeric distributions for terpinen-4-ol [68.5% (+) and 31.5% (−)] and *α*-terpineol [74.2% (+) and 25.8% (−)]. In contrast, greater variation in these ratios was detected among several commercial oils, supporting the usefulness of enantiomeric composition as an indicator of possible adulteration or the addition of constituents from different sources. To date, comparable information has not been reported for *M. viridiflora*, making the enantiomeric characterization performed in the present study relevant. Because terpene biosynthesis is governed by stereoselective enzymatic processes, characteristic enantiomeric ratios may form a more discriminating chemical signature than composition based exclusively on the identity and relative abundance of volatile constituents. Integrating enantioselective gas chromatography with conventional compositional analysis may strengthen the characterization of EOs and provide complementary criteria for their authentication, standardization, and quality control [46].

The *M. viridiflora* EO exhibited limited antibacterial activity under the conditions evaluated. In the present study, inhibition was observed only against *Listeria monocytogenes* and *Enterococcus faecium*, both with MIC values of 4000 µg/mL, whereas no inhibition was detected against *Campylobacter jejuni*, *Escherichia coli* O157, *Pseudomonas aeruginosa*, *Salmonella enterica* serovar Typhimurium, *Enterococcus faecalis*, or *Staphylococcus aureus* at concentrations up to 4000 µg/mL. These results contrast with previous studies of *M. viridiflora* EO, which have reported a broader antibacterial spectrum. Ramanoelina et al. [47] described a particularly pronounced inhibitory effect of *M. viridiflora* EO against Gram-positive bacteria, whereas Fuselli et al. [34] reported inhibition of *Paenibacillus larvae* with an average MIC and MBC of approximately 320 and 590 mg/L, respectively. Sönmez Gürer and Tunç [48] demonstrated antimicrobial activity of *M. viridiflora* EO against *Klebsiella pneumoniae* and *Candida albicans*, although the magnitude of activity varied according to the microorganism and assay employed. The differences among these studies may be related to the chemical composition of oils. For example, the oil evaluated by Fuselli et al. [34] was characterized by terpinen-4-ol (29.09%), *α*-pinene (21.63%), and limonene (17.40%) as major constituents, whereas the present EO was characterized predominantly by 1,8-cineole, together with viridiflorol, limonene, *α*-terpineol, and *α*-pinene. This compositional difference is particularly relevant because terpinen-4-ol has been associated with substantial antimicrobial activity in *Melaleuca* oils. Comparisons within the genus further support this interpretation. *Melaleuca alternifolia* EO, which is typically rich in terpinen-4-ol, has demonstrated inhibitory effects against both Gram-positive and Gram-negative bacteria, including *S. aureus*, *E. coli*, and *P. aeruginosa* [49,50]. The antibacterial action of *Melaleuca* EOs is generally attributed to their hydrophobic terpenoid constituents, which can interact with the bacterial cell envelope, alter membrane permeability, and promote leakage of intracellular components [49,50]. 

The *M. viridiflora* EO showed weak antifungal activity against *Aspergillus niger*, with a MIC of 4000 µg/mL, whereas no inhibition of *Candida albicans* was detected at the highest concentration tested. Information concerning the antifungal activity of *M. viridiflora* EO is relatively limited, but previous studies indicate that its activity can vary substantially according to the microorganism and chemical composition of the oil. Sönmez Gürer and Tunç [48] reported inhibitory activity of *M. viridiflora* EO against *C. albicans* using both disc diffusion and MIC assays, demonstrating that this species can have antifungal potential under proper experimental conditions. The discrepancy with the present results may be associated with differences in the chemical composition of the oils, as well as with methodological differences. At the genus level, stronger antifungal effects have been reported for other *Melaleuca* species. The EO of *M. alternifolia*, for example, has proved antimicrobial activity against filamentous fungi, including *Penicillium italicum* and *P. digitatum* [49]. Similarly, *M. cajuputi* EO showed marked inhibitory effects against toxigenic *Aspergillus flavus*, reducing fungal growth and aflatoxin B1 production at relatively low concentrations [51], this study also demonstrated that *M. cajuputi* EO affected ergosterol biosynthesis, promoted leakage of cellular constituents, and disrupted mitochondrial membrane potential, providing evidence that *Melaleuca* EOs may exert antifungal effects through multiple cellular targets. Differences in antifungal potency among species may again be related to their terpene profiles. The *M. cajuputi* EO evaluated by Chaudhari et al. [51], for example, contained *α*-pinene as its major constituent (49.24%), whereas the present *M. viridiflora* EO was characterized by a predominance of 1,8-cineole. Therefore, although members of the Melaleuca genus may exhibit antifungal properties, the results show that antifungal activity cannot be inferred solely from taxonomic relationships within the genus but is related to the specific chemical profile of each EO.

The antioxidant activity of *M. viridiflora* EO was relatively low when evaluated by radical-scavenging assays. The EO exhibited SC_50_ values of 1191.8 ± 9.4 µg/mL and 7657.5 ± 16.1 µg/mL in the ABTS and DPPH assays, respectively, while Trolox showed substantially greater activity. It was not possible to find studies of antioxidant activity of this essential oil in the existing literature. Direct comparisons with other studies of *Melaleuca* are limited because antioxidant assays and reporting units are not always standardized. Within the genus, considerably stronger antioxidant activity has been reported for some *Melaleuca* EOs. Zhang et al. [49], for example, reported an EC_50_ of 48.35 µg/mL for the DPPH radical-scavenging activity of *M. alternifolia* EO, together with EC_50_ values of 43.71 µg/mL for hydroxyl radical scavenging and an IC_50_ of 135.9 µg/mL in the TBARS assay. Similarly, *M. cajuputi* EO exhibited substantial DPPH and ABTS radical-scavenging activities, with IC_50_ values of 3.16 and 4.29 µL/mL, respectively [51]. These differences among *Melaleuca* species are likely related to their distinct chemical profiles. In *M. alternifolia*, compounds such as *α*-terpinene and *α*-terpinolene have been associated with antioxidant properties. The antioxidant activity of *M. viridiflora* EO may depend on the combined contribution of several constituents rather than on a single highly active compound. The marked difference between the ABTS and DPPH responses also highlights the importance of using complementary antioxidant assays, since the physicochemical characteristics of the radicals and the reaction mechanisms involved can influence the measured activity. Overall, the present results show that *M. viridiflora* EO has limited direct radical-scavenging capacity compared with the stronger antioxidant activity reported for some other *Melaleuca* EOs, although this does not exclude other antioxidant mechanisms that were not evaluated in the present study. The weak DPPH and ABTS radical-scavenging activity observed for *M. viridiflora* essential oil may be associated with its chemical profile, characterized by a predominance of monoterpenes and oxygenated monoterpenes such as 1,8-cineole [52], together with a low abundance of phenolic constituents. Phenolic compounds generally exhibit stronger radical-scavenging properties because their hydroxyl groups facilitate hydrogen-atom or electron donation and stabilize the resulting radical species. In contrast, 1,8-cineole and several common monoterpenes exhibit comparatively limited radical-scavenging capacity, especially in DPPH-based assays [53].

According to the classification scale (high potency of IC_50_ < 20 µg/mL, moderate potency of 20 < IC_50_ < 200 µg/mL, and low potency of 200 < IC_50_ < 1000 µg/mL) proposed by Santos et al. [54] the EO of *M. viridiflora* exhibited moderate acetylcholinesterase (AChE) inhibitory activity, with an IC_50_ value of 65.98 ± 1.0 µg/mL. It was not possible to find previous studies on the AChE inhibitory activity of *M. viridiflora* EO. For major compound 1,8 cineole, Perry et al. [55] reported an IC_50_ of 0.67 mM (103.3 µg/mL) against human erythrocyte AChE, while Savelev et al. [56] found an CI_50_ of 0.06 ± 0.01 mg/mL (60 ± 10 µg/mL) and Pavarino et al. [57] reported an IC_50_ of 14.1 ± 0.556 µg/mL. Few studies on the anticholinesterase activity of EOs from species of this genus are reported in the literature. In *M. alternifolia*, AChE inhibition has been reported, with an IC_50_ of 51.2 µg/mL for tea tree oil [58], 0.05–0.11 µL/mL in commercial samples [59], and 100% AChE inhibition in an assay with Anisakis simplex at 100 µL/mL [60]. Petrachaianan et al. [61] reported 21.18% AChE inhibition produced by *M. cajuputi* essential oil. The difference may be related to the distinct chemical profiles of the oils. The *M. alternifolia* EO examined by Miyazawa et al. [58] contained terpinen-4-ol (35.6%), γ-terpinene (19.4%), and *α*-terpinene (8.3%) as its major constituents. Although the contribution of individual constituents to the AChE inhibition observed here cannot be established from the current data, terpenoids present in EO may interact with enzymes through different mechanisms (competitive, non-competitive, mixed and reversible/uncompetitive inhibitions), and the resulting activity can reflect additive or synergistic interactions among several constituents [62]. Indeed, studies with individual essential oil constituents have demonstrated that compounds such as *α*-pinene can affect acetylcholinesterase activity, supporting the possibility that monoterpenes contribute to the enzyme-inhibitory effects of complex EOs [63].

## 4. Materials and Methods

### 4.1. Materials

The aliphatic hydrocarbon standards used to generate the GC-FID calibration curve were supplied by ChemService (West Chester, PA, USA), and helium was obtained from INDURA (Quito, Ecuador). Culture media used for antimicrobial susceptibility testing, including Sabouraud dextrose broth (SDB), fluid thioglycollate medium, Mueller–Hinton broth, and Mueller–Hinton II broth, were sourced from DIPCO (Quito, Ecuador). Sigma-Aldrich (St. Louis, MO, USA) provided phosphate-buffered saline (PBS), Tris-HCl, and the reagents required for the biological assays and sample preparation: 2′-azinobis(3-ethylbenzothiazoline-6-sulfonic acid) (ABTS), 2,2-diphenyl-1-picrylhydrazyl (DPPH), 2, 5,5′-dithiobis(2-nitrobenzoic acid) (DTNB), acetylcholinesterase (AChE), acetylthiocholine (AcSCh), butylated hydroxytoluene (BHT), dichloromethane (DCM), donepezil, dimethyl sulfoxide (DMSO), methanol (MeOH), magnesium chloride hexahydrate, anhydrous sodium sulfate, and Trolox. All reagents were of analytical grade and were used without additional purification. 

### 4.2. Plant Material

Leaves of *M. viridiflora* were collected on three separate occasions at one-week intervals from different individual trees within a cultivated plantation located in the vicinity of Guayllabamba Parish, Quito Canton, Pichincha Province, Ecuador. Guayllabamba has a dry mesothermal climate that can be classified as Cwb (subtropical highland climate with a dry winter and warm summer). The sampling site corresponded to a valley situated at 0°04′43″ S and 78°20′59″ W, at an altitude of 2171 m above sea level. After collection, the plant material was carefully stored and transported in sealed plastic containers. During both the collection and transportation processes, the environmental conditions were kept at a pressure of approximately 79 kPa and a temperature ranging between 18 and 20 °C.

### 4.3. Postharvest Treatments

After the collection, the leaves were immediately subjected to postharvest treatment. This treatment involved the removal of foreign or deteriorated leaf samples.

### 4.4. Essential Oil Isolation

The whole *M. viridiflora* leaves were extracted by steam distillation using a distiller (200 L, local construction). The extraction process begins by placing the fresh leaves in the distiller and passing steam through them; the steam was previously obtained in a boiler [64]. The distillation process was maintained for 3 h, with the extraction time measured from the appearance of the first distillate drop. After condensation of the water and EO vapors, the oil phase was recovered by decantation [64] and dehydrated over anhydrous sodium sulfate. The resulting EO was transferred to airtight amber vials and preserved at 4 °C until further analysis.

### 4.5. Determination of the Physical Properties of the Essential Oil

Three objective physical parameters of the EO were evaluated, namely density, refractive index and optical rotation, together with one subjective characteristic, color. The density was determined according to the AFNOR NF T 75-111 standard, which is equivalent to ISO 279:1998 [65], using an analytical balance (Mettler AC 100, Mettler Toledo, Columbus, OH, USA). The refractive index was measured following the AFNOR NF T 75-112 protocol, comparable to ISO 280:1998 [66], with an ABBE refractometer (BOECO, Hamburg, Germany). Optical rotation was assessed in accordance with ISO 592:1998 [67] employing an automatic polarimeter (Mrc-P810, MRC, Holon, Israel). In addition, the color of the EO, considered a subjective attribute, was determined through digital analysis using the PineTools platform (website https://pinetools.com/, accessed on 13 December 2025), where an image of the sample placed on a white background was uploaded. All determinations were performed under controlled conditions at a constant temperature of 20 °C.

### 4.6. Identification of Essential Oil Compounds

#### 4.6.1. Quantitative and Qualitative Analysis

The volatile constituents of the essential oil (EO) obtained from *M. viridiflora* leaves were identified by gas chromatography coupled to mass spectrometry (GC–MS). Analyses were performed on a TRACE™ 1310 gas chromatograph coupled to an ISQ 7000 quadrupole mass spectrometer and equipped with an AI 1310 autosampler (Thermo Scientific, Waltham, MA, USA). Chromeleon 7.2 software was used for chromatographic data acquisition and processing, and mass spectral comparisons were supported by the NIST 17 and ADMIS 2.7 libraries.

Separation was performed on a 5% phenyl-methylpolysiloxane capillary column (30 m × 0.25 mm i.d., 0.25 µm film thickness). Before analysis, the EO was diluted 1:100 (*v*/*v*) with dichloromethane, and 1 µL of the resulting solution was injected using a 1:50 split ratio. Helium served as the carrier gas at a flow rate of 0.9 mL/min, corresponding to an average linear velocity of 34 cm/s. The injector temperature was maintained at 230 °C. The oven was initially set at 50 °C for 3 min, subsequently increased to 230 °C at 3 °C/min, and maintained at the final temperature for 3 min, giving a total chromatographic run of 66 min.

Mass spectra were acquired under electron-impact ionization (EI) at 70 eV over an *m*/*z* range of 40–350, using a scan rate of 2 scans/s and an electron multiplier voltage of 1600 V. Retention indices (RI) were determined from the retention times of a homologous series of n-alkanes analyzed under the same chromatographic conditions according to Equation (1) [68]. Compound identification was accomplished by comparing the experimentally determined RI values and mass spectra with published reference data [69] and spectral-library information.

(1) $$\mathrm{RI}=100C+100\frac{\left(t_{R}\left(x\right)-t_{R}\left(n\right)\right)}{\left(t_{R}\left(n+1\right)-t_{R}\left(n\right)\right)}$$

where *n* denotes the number of carbon atoms in the n-alkane eluting immediately before the analyte, (t_R_(x)) represents the retention time of the compound under investigation, and (t_R_(*n*)) and (t_R_(*n*+1)) are the retention times of the n-alkanes eluting immediately before and after the analyte, respectively.

#### 4.6.2. Qualitative Analysis

Quantitative analysis of the *M. viridiflora* leaf EO was performed by gas chromatography coupled to a flame ionization detector (GC–FID) using a TRACE™ 1310 gas chromatograph equipped with an AI 1310 autosampler (Thermo Scientific, Waltham, MA, USA). Chromatographic separation was carried out on a 5% phenyl-methylpolysiloxane capillary column (30 m × 0.25 mm i.d., 0.25 µm film thickness). The EO was diluted in dichloromethane at a 1:100 (*v*/*v*) ratio, and a 1 µL aliquot was injected in split mode (1:50).

Helium was employed as the carrier gas at a flow rate of 1.0 mL/min, corresponding to an average linear velocity of 25 cm/s. Both the injector and FID temperatures were maintained at 230 °C. The oven temperature was programmed from 50 °C (3 min) to 230 °C at a heating rate of 3 °C/min, followed by a 3 min isothermal period at 230 °C. The relative abundance of each constituent was calculated from its integrated GC–FID peak area and expressed as a percentage of the total chromatographic area, without applying response correction factors.

### 4.7. Enantioselective Analysis

The chromatographic and MS operating conditions, including the split ratio, helium flow, injector temperature, oven program, and MS parameters, were maintained as described above for the qualitative GC–MS analysis. Enantiomer elution order and absolute configuration assignments were established according to the technical specifications provided for each chiral column. Enantiomeric distribution (ED) was determined from the relative integrated peak areas of each enantiomeric pair. Enantiomeric excess (e.e.) was subsequently calculated from the difference between the proportions of the major and minor enantiomers according to: e.e. (%) = % major enantiomer − % minor enantiomer.

The enantiomeric profile of the *M. viridiflora* leaf EO was investigated by chiral GC–MS using the chromatographic system, data-processing software, spectral libraries, autosampler, and mass spectrometric detector specified above for qualitative analysis. Enantiomer separation was examined on two capillary columns (30 m × 0.25 mm i.d., 0.25 μm film thickness) with different β-cyclodextrin-based stationary phases. A MEGA-DEX DET-Beta column (Mega, Legnano, Italy) containing diethyl tert-butyldimethylsilyl-β-cyclodextrin and a MEGA-DEX DAC-Beta column (Mega, Legnano, Italy) coated with diacetyl tert-butyldimethylsilyl-β-cyclodextrin were used.

### 4.8. Determination of Antimicrobial Activity

The antimicrobial activity of *M. viridiflora* EO was assessed against a panel of bacterial and fungal reference strains using the broth microdilution method described by Valarezo et al. [70]. The bacterial panel included the Gram-negative bacilli *Campylobacter jejuni* (ATCC 33560), *Escherichia coli* O157 (ATCC 43888), *Pseudomonas aeruginosa* (ATCC 10145), and *Salmonella enterica* subsp. *enterica* serovar Typhimurium WDCM 00031 (derived from ATCC 14028); the Gram-positive cocci *Enterococcus faecalis* (ATCC 19433), *Enterococcus faecium* (ATCC 27270), and *Staphylococcus aureus* (ATCC 25923); and the Gram-positive bacillus *Listeria monocytogenes* (ATCC 19115). Antifungal activity was evaluated against *Aspergillus niger* (ATCC 6275) and *Candida albicans* (ATCC 10231).

Assays were conducted in 96-well microplates using two-fold serial dilutions of the EO over a concentration range of 15.62–4000 µg/mL. For *C. jejuni*, concentrations ranged from 31.25 to 4000 µg/mL [71]. For the antifungal assay, the EO was prepared in SDB containing the fungal inoculum, with a final spore concentration of 5 × 10^4^ spores/mL. Ciprofloxacin was used as the positive control for Gram-negative bacilli and *L. monocytogenes*, whereas ampicillin was used for Gram-positive cocci. Amphotericin B served as the positive control for the fungal strains, and 5% DMSO was used as the negative control. Antimicrobial activity was expressed as the minimum inhibitory concentration (MIC), defined as the lowest EO concentration at which visible microbial growth was inhibited after incubation.

### 4.9. Determination of Antioxidant Activity

The antioxidant capacity of the *M. viridiflora* EO was evaluated using the ABTS and DPPH radical-scavenging assays following the methodology described by Valarezo et al. [72]. Both assays were performed by a colorimetric approach using a Genesys 10S UV–Vis spectrophotometer (Thermo Fisher Scientific, Waltham, MA, USA), with Trolox and methanol serving as the positive and negative controls, respectively. The maximum EO concentration tested was 10,000 µg/mL.

For the ABTS assay, antioxidant activity was determined from the decrease in absorbance of the ABTS radical cation (ABTS•^+^) at 734 nm, whereas DPPH radical (DPPH•) scavenging was monitored at 515 nm. In both assays, the radical-scavenging capacity was expressed as the half-scavenging concentration (SC_50_), defined as the EO concentration required to produce 50% inhibition. SC_50_ values were obtained from dose–response curves by plotting the percentage of radical inhibition against the logarithm (log_10_) of EO concentration.

For the ABTS assay, trolox equivalent antioxidant capacity (TEAC) was additionally determined from the ratio between the SC_50_ values of trolox and the EO. TEAC values were subsequently reported as µmol trolox equivalents per gram of essential oil (µmol TE/g).

### 4.10. Determination of Anticholinesterase Activity

Acetylcholinesterase (AChE) inhibitory activity of the *M. viridiflora* EO was determined using the Ellman colorimetric method [73], with the experimental conditions described by Valarezo et al. [74]. Measurements were performed in a 96-well microplate using an EPOCH 2 spectrophotometer (BioTek, Winooski, VT, USA). Each reaction mixture contained 40 µL of Buffer C, 20 µL of EO solution prepared in methanol (10, 100, or 1000 µg/mL), 20 µL of acetylthiocholine (AcSCh), and 100 µL of Ellman’s reagent (DTNB). After pre-incubation at 25 °C for 3 min, the enzymatic reaction was initiated by adding 20 µL of AChE (0.5 U/mL; 137 U/mg solid), and absorbance changes were monitored at 412 nm. The half-maximal inhibitory concentration (IC_50_) was estimated by nonlinear regression of the normalized response (initial reaction rate) against the logarithm of inhibitor concentration using a variable-slope model. Donepezil and methanol were employed as the positive and negative controls, respectively.

### 4.11. Statistical Analysis

All experimental data were recorded in Microsoft Excel spreadsheets and subsequently processed using Minitab 17 (version 17.1.0, Minitab LLC., State College, PA, USA) to obtain descriptive statistics, including measures of central tendency and standard deviation. The antioxidant and anticholinesterase activity results were evaluated using GraphPad Prism (version 6.0, GraphPad Software Inc., San Diego, CA, USA).

All experimental procedures, including collection of plant material, essential oil extraction, physicochemical characterization, the identification of EO constituents and enantiomeric analysis were carried out in triplicate. In contrast, antioxidant evaluation, anticholinesterase assays, and antimicrobial tests were performed with nine replicates (three samples × three replicates) to ensure the reliability and reproducibility of the results.

## 5. Conclusions

This study provides the first analysis of the chemical composition and biological activities of the essential oil isolated from leaves of *Melaleuca viridiflora* Sm. cultivated in the Americas. This is the first report of physical properties, enantiomeric distribution, and antioxidant and antienzymatic activity of this essential oil. It is determined that the physicochemical properties and chemical composition of the essential oil are consistent with a volatile oil rich in terpenes. The chemical profile was characterized predominantly by oxygenated monoterpenes (45%) and monoterpenes hydrocarbons (27%), with 1,8-cineole (36%) as the major constituent. 

The biological assays indicated that *M. viridiflora* EO exhibited limited antibacterial and antifungal activity under the experimental conditions evaluated, with activity restricted to selected microorganisms. Its relatively weak radical-scavenging capacity further suggests that the oil should not be considered a strong direct antioxidant based solely on the DPPH and ABTS assays. In contrast, the reported acetylcholinesterase-inhibitory activity (66 µg/mL) indicates a potentially relevant biological property that merits further attention.

These findings contribute to the currently limited chemical and biological knowledge of *M. viridiflora* essential oil and provide a basis for its differentiation from oils obtained from other Melaleuca species. Furthermore, these results contribute to the knowledge of aromatic flora biodiversity of Ecuador. 

Future studies should investigate the influence of geographic origin, seasonal and phenological variation, and extraction conditions on the chemical profile and yield of *M. viridiflora* essential oil. In terms of biological aspects, further in vivo studies could be conducted, for example, to assess its anti-inflammatory or insecticidal activity, to better evaluate its potential applications.

## Figures and Tables

**Table 1:** Physical properties of the essential oil from leaves of *Melaleuca viridiflora*.

	***M. viridiflora* Leaves EO**	
	**Mean**	
Density, *ρ* (g/cm^3^)	0.8933 (±0.0057)	
Refractive index, *n*^20^	1.4733 (±0.0005)	
Optical rotation, [*α*] (°)	−6.48 (±0.10)	
Color		
Subjective	Moderate yellow	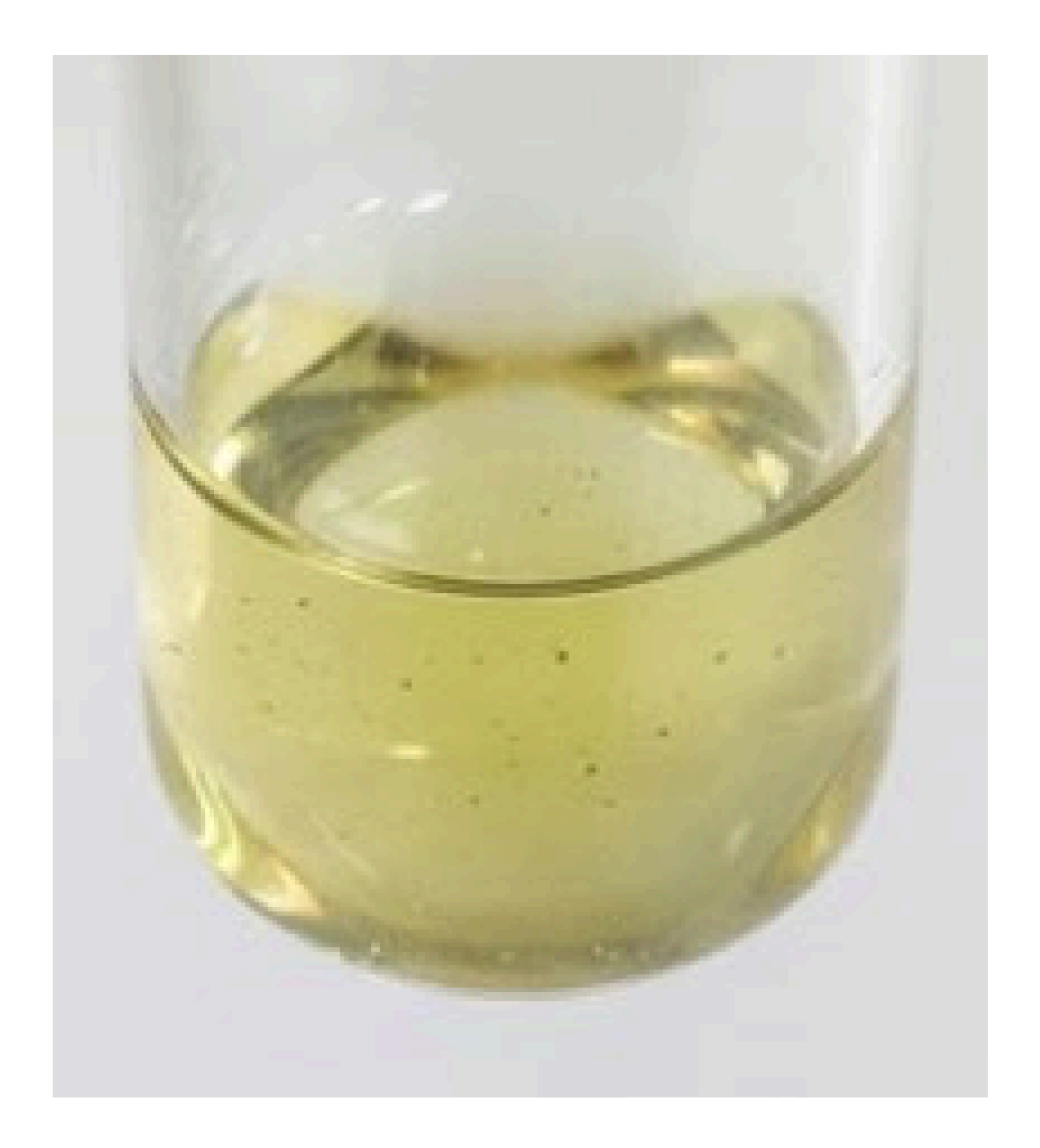
RGB color values	185, 157, 50	
CMYK color values	0%, 15%, 73%, 27%	
Hex Color Codes	#B99D32	

SD: standard deviation.

**Table 2:** Chemical composition of essential oil from leaves of *Melaleuca viridiflora*.

CN	RT	Compound	RIC	RIR	RA (%)	SD	CG	CF	MM (Da)
1	9.05	*α*-Thujene	922	924	0.25	0.00	MH	C_10_H_16_	136.13
2	9.38	*α*-Pinene	929	932	7.42	0.11	MH	C_10_H_16_	136.13
3	10.14	Camphene	945	946	0.17	0.00	MH	C_10_H_16_	136.13
4	11.17	Sabinene	966	969	0.07	0.01	MH	C_10_H_16_	136.13
5	11.40	*β*-Pinene	971	974	4.58	0.04	MH	C_10_H_16_	136.13
6	11.89	Myrcene	981	988	0.70	0.01	MH	C_10_H_16_	136.13
7	12.74	*δ*-2-Carene	999	1001	0.65	0.01	MH	C_10_H_16_	136.13
8	13.23	*α*-Terpinene	1009	1014	0.32	0.01	MH	C_10_H_16_	136.13
9	13.70	*ρ*-Cymene	1019	1020	0.68	0.02	MH	C_10_H_14_	134.11
10	13.85	Limonene	1022	1024	11.04	0.06	MH	C_10_H_16_	136.13
11	13.96	*β*-Phellandrene	1024	1025	0.24	0.00	MH	C_10_H_16_	136.13
12	14.12	1,8-Cineole	1027	1026	35.66	0.37	OM	C_10_H_18_O	154.14
13	14.65	(*Z*)-*β*-Ocimene	1038	1032	0.13	0.00	MH	C_10_H_16_	136.13
14	15.26	*γ*-Terpinene	1051	1054	0.87	0.01	MH	C_10_H_16_	136.13
15	16.57	Terpinolene	1078	1086	0.29	0.00	MH	C_10_H_16_	136.13
16	17.45	Linalool	1096	1095	0.19	0.01	OM	C_10_H_18_O	154.14
17	17.64	*trans*-Sabinene hydrate (IPP vs. OH)	1100	1098	0.03	0.01	OM	C_10_H_18_O	154.14
18	21.09	*δ*-Terpineol	1171	1162	0.05	0.00	OM	C_10_H_18_O	154.14
19	21.49	Terpinen-4-ol	1179	1174	0.93	0.02	OM	C_10_H_18_O	154.14
20	22.30	*α*-Terpineol	1196	1186	7.89	0.71	OM	C_10_H_18_O	154.14
21	23.66	Citronellol	1225	1223	0.04	0.01	OM	C_10_H_20_O	156.15
22	23.95	(*E*)-Ocimenone	1231	1235	0.06	0.00	OM	C_10_H_14_O	150.10
23	24.79	Geraniol	1249	1249	0.41	0.02	OM	C_10_H_18_O	154.14
24	26.62	(*E*)-Anethole	1288	1282	0.07	0.00	OC	C_10_H_12_O	137.00
25	28.06	Myrtenyl acetate	1320	1324	0.13	0.00	OC	C_12_H_18_O_2_	194.13
26	31.50	*α*-Gurjunene	1398	1409	0.07	0.01	SH	C_15_H_24_	204.19
27	32.11	(*E*)-Caryophyllene	1412	1417	1.26	0.03	SH	C_15_H_24_	204.19
28	32.92	*α*-Guaiene	1432	1437	0.16	0.01	SH	C_15_H_24_	204.19
29	33.23	Aromadendrene	1439	1439	0.11	0.13	SH	C_15_H_24_	204.19
30	33.68	*α*-Humulene	1450	1452	0.27	0.01	SH	C_15_H_24_	204.19
31	33.86	*allo*-Aromadendrene	1454	1458	0.29	0.01	SH	C_15_H_24_	204.19
32	35.15	Viridiflorene	1484	1496	0.37	0.01	SH	C_15_H_24_	204.19
33	35.39	Bicyclogermacrene	1490	1500	0.22	0.01	SH	C_15_H_24_	204.19
34	36.11	*γ*-Cadinene	1508	1513	0.07	0.00	SH	C_15_H_24_	204.19
35	37.68	Elemol	1547	1548	0.07	0.01	OS	C_15_H_26_O	222.20
36	38.56	Palustrol	1569	1567	0.36	0.01	OS	C_15_H_26_O	222.20
37	38.90	Spathulenol	1578	1577	0.18	0.01	OS	C_15_H_24_O	220.18
38	39.09	Caryophyllene oxide	1582	1582	0.64	0.02	OS	C_15_H_24_O	220.18
39	39.24	Globulol	1586	1590	1.41	0.05	OS	C_15_H_26_O	222.20
40	39.61	Viridiflorol	1595	1592	14.20	0.12	OS	C_15_H_26_O	222.20
41	39.99	Ledol	1605	1602	4.08	0.05	OS	C_15_H_26_O	222.20
42	40.23	Humulene epoxide II	1612	1608	0.11	0.01	OS	C_15_H_24_O	220.18
43	40.32	1,10-di-epi-Cubenol	1614	1618	0.03	0.00	OS	C_15_H_26_O	222.20
44	40.82	1-*epi*-Cubenol	1627	1627	0.09	0.00	OS	C_15_H_26_O	222.20
45	41.02	*epi*-*α*-Cadinol	1633	1638	0.39	0.04	OS	C_15_H_26_O	222.20
46	41.41	Hinesol	1643	1640	0.25	0.02	OS	C_15_H_26_O	222.20
47	41.51	*epi*-*α*-Muurolol	1646	1640	0.10	0.04	OS	C_15_H_26_O	222.20
48	41.99	*α*-Eudesmol	1658	1652	1.18	0.03	OS	C_15_H_26_O	222.20
49	42.29	Bulnesol	1666	1670	0.21	0.01	OS	C_15_H_26_O	222.20
50	43.47	Shyobunol	1698	1688	0.08	0.00	OS	C_15_H_26_O	222.20
Total identified					99.07%				

CN: compound number, assigned according to their elution order; RT: retention time; RIC: calculated retention indices; RIR: retention index based on the literature; RA: relative abundance; SD: standard deviation; CG: compound group; MH: monoterpene hydrocarbons; OM: oxygenated monoterpenes; SH: sesquiterpene hydrocarbons; OS: oxygenated sesquiterpene; OC: other compounds; CF: chemical formula; MM: monoisotopic mass.

**Table 3:** Enantiomeric distribution of the essential oil from leaves of *Melaleuca viridiflora*.

RT	Enantiomers	RI	ED (%)	e.e. (%)	Rs
MEGA-DEX DET Beta column					
3.79	(+)-(1*R*,5*R*), (−)-(1*S*,5*S*), *α*-Pinene	937	1.74	96.61	1.2
3.87		941	98.26		
5.18	(+)-(1*R*,5*R*)-*β*-Pinene	993	2.67	94.67	1.1
5.21	(−)-(1*S*,5*S*)-*β*-Pinene	994	97.33		
7.46	(−)-(4*S*), (+)-(4*R*), Limonene	1055	77.92	55.85	1.6
7.70		1059	22.08		
20.98	(−)-(4*S*)-*α*-Terpineol	1297	32.39	35.23	1.5
21.38	(+)-(4*R*)-*α*-Terpineol	1303	67.61		
MEGA-DEX DAC Beta column					
24.32	(+)-(3*S*), (−)-(3*R*), Linalool	1290	75.57	51.15	1.4
24.46		1292	24.43		
30.30	(+)-(4*R*), (−)-(4*S*), *α*-Terpineol	1385	46.55	6.90	1.3
30.57		1389	53.45		

RT: retention time; RI: retention index; ED: enantiomeric distribution; e.e.: enantiomeric excess; Rs: resolution.

**Table 4:** Antibacterial activity of essential oil from leaves of *Melaleuca viridiflora*.

Microorganism	*M*. *viridiflora* EO	Positive Control *	Negative Control
	MIC (µg/mL)		
Gram-negative bacilli			
*Campylobacter jejuni* (ATCC 33560)	>4000	1.56	+
*Escherichia coli* O157:H7 (ATCC 43888)	>4000	1.56	+
*Pseudomonas aeruginosa* (ATCC 10145)	>4000	0.39	+
*Salmonella enterica* subs enterica serovar *Typhimurium* WDCM 00031, derived (ATCC 14028)	>4000	0.39	+
Gram-positive bacillus			
*Listeria monocytogenes* (ATCC 19115)	4000	1.56	+
Gram-positive cocci			
*Enterococcus faecalis* (ATCC 19433)	>4000	0.78	+
*Enterococcus faecium* (ATCC 27270)	4000	0.39	+
*Staphylococcus aureus* (ATCC 25923)	>4000	0.39	+

*: Ciprofloxacin for Gram-positive bacillus and Gram-negative bacilli and ampicillin for Gram-positive cocci; MIC: minimum inhibitory concentration; +: normal growth.

**Table 5:** Antimicrobial activity of essential oil from leaves of *Melaleuca viridiflora*.

Microorganism	*M*. *viridiflora* EO	Positive Control *	Negative Control
	MIC (µg/mL)		
Fungus			
*Aspergillus niger* (ATCC 6275)	4000	0.098	+
Yeast			
*Candida albicans* (ATCC 10231)	>4000	0.098	+

*: Amphotericin B; MIC: minimum inhibitory concentration; +: normal growth.

**Table 6:** Antioxidant activity of essential oil from leaves of *Melaleuca viridiflora*.

Sample	ABTS SC_50_ (µg/mL)	DPPH SC_50_ (µg/mL)	TEAC (µM TE/g)
*M. viridiflora* EO	1191.8 ± 9.4	7657.5 ± 16.1	34.6 ± 0.62
Trolox	7.28 ± 0.28	8.89 ± 0.25	

ABTS: 2,2′-azinobis(3-ethylbenzothiazoline-6-sulfonic acid); DPPH: 2,2-diphenyl-1-picrylhydrazyl; TEAC: Trolox equivalent antioxidant capacity; SC_50_: half maximal scavenging capacity: TE: trolox equivalent.

## Data Availability

The original contributions presented in this study are included in the article. Further inquiries can be directed to the corresponding author.
